# Deep‐water cirripedes colonizing dead shells of the cephalopod *Nautilus macromphalus* from New Caledonian waters

**DOI:** 10.1111/1749-4877.12389

**Published:** 2019-11-25

**Authors:** John BUCKERIDGE, Tomáš KOČÍ, Ján SCHLÖGL, Adam TOMAŠOVÝCH, Martina KOČOVÁ VESELSKÁ

**Affiliations:** ^1^ Earth & Oceanic Systems Group RMIT University Melbourne Victoria Australia; ^2^ Ivančická 581, Prague, Czech Republic and Palaeontological Department, Natural History Museum, National Museum, Prague, Czech Republic; ^3^ Department of Geology and Paleontology Comenius University in Bratislava Bratislava Slovakia; ^4^ Institute of Earth Sciences Slovak Academy of Sciences Bratislava Slovakia; ^5^ Department of Paleobiology and Paleoecology Institute of Geology of the Czech Academy of Sciences Prague Czech Republic; ^6^ Museums Victoria Melbourne Victoria Australia; ^7^ Faculty of Science Charles University in Prague Prague Czech Republic

**Keywords:** *Anellusichnus ellipticus* isp. nov., cirripedes, epibionts, *Hexelasma velutinum*, *Metaverruca recta*

## Abstract

Fossil cephalopods are frequently encrusted by epibionts; however, determining whether encrustation occurred prior to or post‐mortem to the host, and whether the final environment of deposition corresponds to the habitat of encrustation is complex. The present paper describes cirripede epibionts, their calcareous bases and their attachment scars on 6 post‐mortem shells of *Nautilus macromphalus*, collected from deep water off New Caledonia. The cirripedes have left both cemented calcareous bases of *Hexelasma* and scars associated with bioerosion and discoloration produced by verrucomorph barnacles. Live cirripedes included a *Metaverruca recta*, with articulated opercular plates and organic tissue (on a shell that had been exposed on the sea floor for at least 150 years), and specimens of *Hexelasma velutinum*, one of which was partly attached to an internal surface of a shell. The disposition of verrucomorphs indicates that most *Nautilus* shells were colonized post‐mortem rather than during a floating stage. However, as cirripedes are known to have colonized living *Nautilus*, some *Hexelasma*, preserved only as calcareous eroded bases, may represent specimens that settled on a living *Nautilus*. The degree of bioerosion and discoloration induced by verrucomorph barnacles varies according to the surface preservation of *Nautilus* shells, with deeper and discolored traces preserved on old and degraded shells. Traces made by verrucomorphs described here are ellipsoidal and a new ichnotaxon, *Anellusichnus ellipticus*, is proposed to accommodate them. Importantly, verrucomorphs and other cirripede taxa with membranous bases that were attached to pristine shells may not leave any substantial scars, and, thus, will be difficult to detect in the fossil record.

## INTRODUCTION

Cephalopod encrusters with preservable skeletal remains are informative about the life habits, habitats and ecology of hosts and encrusters (Seilacher 1960; Baird

*et al*. 1989; Keupp *et al.* 1999; Lukeneder 2008; Zell *et al*. 2014; Hautmann 2017). They may also represent post-mortem colonization of cephalopod shells during their floating phase or when exposed on the seafloor after their death (Seilacher 1968; Donovan 1989; Rakús & Žítt 1993; Klug & Korn 2001; Landman *et al*. 2010; Wyse Jackson & Key 2014; Luci & Cichowolski 2014; Mironenko 2015; Yacobucci 2018). Dead cephalopod shells and shells of other large molluscs provide relatively stable substrates for benthic islands (Rakociński 2011), and thus, can have significant effects on the composition of encrusting and hard-substrate communities (Brett 1988; Nebelsick *et al*. 1997). However, some encrusters do not have any preservation potential (Lescinsky 1993) and late-succession colonizers can erase the former record of skeletal encrusters through destructive processes (Rasmussen & Brett 1985). Therefore, studies analyzing encrustation patterns on modern cephalopod shells can be helpful in constraining our inferences about ecology and evolution of encrusters preserved in the fossil record (Ritterbush *et al.* 2014).

The complexity of encrusting communities preserved on dead cephalopod shells can be high. In the first instance, their shells may be colonized during life at various depths that can be inhabited by different larval pools, although colonization by encrusters can be enhanced during their resting periods at approximately 200 m when they retain neutral buoyancy (Dunstan *et al*. 2011). There are numerous records of infestation of living *Nautilus*, demonstrating that this is certainly possible (e.g. Aurivillius 1892, 1894; Seilacher 1982; Landman 1983). Second, post-mortem *Nautilus* shells may be colonized when floating into or near surface waters. Third, empty shells may become encrusted after sinking to the sea floor. Although the periostracum can limit bioerosion and epibiont settlement on *Nautilus* shells (Seuss *et al*. 2015), various encrusters, including foraminifera, barnacles, polychaetes, bryozoans, oysters, corals and sponges, have been recorded on shells of living *Nautilus* (Hamada 1983; Landmann 1983; Suzuki & Hayasaka 1988; Landmann *et al*. 2010). However, the degree of encrustation can be geographically variable. Hamada (1983) contrasted living *Nautilus* from Philippines waters, which were encrusted by bryozoans, cirripedes (the balanomorph *Amphibalanus* and a lepadomorph, the latter being tentatively identified as *Conchoderma*) and foraminifera, with *Nautilus* from New Caledonian waters, which are colonized infrequently, generally by small lepadid barnacles and serpulid worms (Hamada 1983; Suzuki & Hayasaka 1988). Landman *et al*. (2014) records approximately 7000-year-old death assemblages of *Nautilus macromphalus* Sowerby, 1849 in a karst system, now at 40-m water depth without any epibionts. *Nautilus* shells collected on beaches (backshore) and at 1–3-m water depth in lagoons at Lifou Islands were primarily encrusted by cyanobacteria and calcareous and non-calcareous algae (Mapes *et al*. 2010a).

In this paper, we describe the assemblage of barnacles attached to 6 empty shells of *Nautilus* retrieved from epibathyal and mesobathyal waters off New Caledonia (Fig. 1; Tomašových *et al*. 2016). We discuss the timing of colonization of shells by barnacles; that is, whether it occurred during life or post-mortem, and whether the colonization occurred during the floating stage or on the sea floor. Finally, we focus on the preservation state of their attachment bases and scars. Our observations will improve the interpretation of cirripede traces in the fossil record.

Cirripedes from waters off New Caledonia in the Southwest Pacific have been described by Buckeridge (1994, 1997) and Jones (2000, 2007). The collection described here augments these observations with new records of verrucomorphs and balanomorphs as encrusters on cephalopods. Although for the most part, these cirripedes are incomplete, (being represented by attachment scars and calcareous bases), sufficient intact shells permit species-level identification.

## MATERIALS

Twenty-one post-mortem shells of *N. macromphalus* were sampled with beam trawls and dredges along the south–southeastern margin of New Caledonia (off Lifou

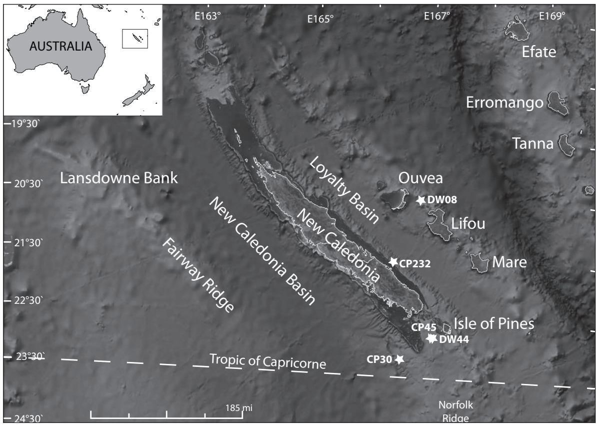

**Figure 1** Geographical distribution of sample sites that provided post-mortem *Nautilus macromphalus* shells with cirripede epibionts (after Tomašových *et al.* 2017)

Island, off Isle of Pines and off Great Southern Reef), at Lansdowne Bank (located between New Caledonia and the Chesterfield Islands) and at the Antigonia Seamount (Fig. 1) in 1985 and 1987 in the course of Biocal and Biogeocal campaigns (De Forges 1990; Tomašových *et al*. 2016, 2017). All specimens, including types, are held in the collections of the Université de Bourgogne in Dijon and are curated with the prefix "UBGD" (e.g. as "UBGD 30750"). From this material, 6 shells with attached cirripedes and/or cirripede bases were recovered. Specimen UBGD 30750 (from station DW 08) bears 2 attached barnacles with compartmental and opercular plates. Specimen UBGD 30735 (station CP 45) has an attached balanomorph shell that does not possess any opercula. Four other specimens, UBGD 30739, UBGD 30741, UBGD 30740 and UBGD 30748 (from stations CP 30, CP 45, CP 232 and DW 44), possess circular barnacle attachment scars and/or non-porous calcareous barnacle bases. Four shells (from stations DW 08, CP 45 and DW 44) were collected from the epibathyal zone (<750 m) and 2 (from stations CP 30 and CP 232) from the mesobathyal zone (>750 m). Geographical and field sampling data are summarized in Table 1. The shell UBGD 30740 from stn CP 232 was dated with $^{14}C$ (Tomašových *et al*. 2016); the other 5 shells with cirripede epizoans were dated by radiocarbon-calibrated amino acid racemisation, using the D/L of glutamic acid in the prismatic layer (Table 1, Tomašových *et al*. 2016).

## GEOGRAPHICAL AND ENVIRONMENTAL SETTINGS

The 6 shells with barnacles, barnacle attachment scars and/or calcareous bases were collected south (stns CP 30, CP 45/1, CP 45/2 and DW 44/2) and east (stn CP 232) of New Caledonia and northwest of Lifou Island (stn DW 08). Water depths ranged between 430 and 1140 m (see Table 1). Seawater temperatures declined from 12–13 °C at 430 m to 7 °C at 700 m in the epibathyal zone, to 3–4 °C at 1140 m in the mesobathyal zone north of Lifou Island (CALSUB, Roux 1991). Water temperatures ranged between 5 and 6 °C at depths of 760–790 m east of Thio (specimen UBGD 30740, Roux 1991).

## METHODOLOGY

Shells with epibionts were examined under a Bresser (20× to 40×) (T. K.) light microscope and photographed under an Olympus DP70 light microscope with a Canon EOS 6D camera at the National Museum in Prague. Shell microstructures were examined at the Institute of Geology and Palaeontology, Faculty of Science, Charles University in Prague by scanning electron microscope (SEM) JEOL-6380LV. Plates were prepared using Corel Graphic Suite X4 and Adobe Photoshop CS5.

## SYSTEMATICS

**Superorder Thoracica Darwin, 1854**

**Order Verrucomorpha Pilsbry, 1916**

**Family Verrucidae Darwin, 1854 (amend. Newman & Hessler, 1989)**

**Genus *Metaverruca* Pilsbry, 1916**

***Metaverruca recta* (Aurivillius, 1898)**

(Fig. 2a, a2)

*Verruca recta* Aurivillius, 1898: 195.

*Verruca sculpta* Aurivillius, 1898: 197.

*Verruca halotheca* Pilsbry, 1916: 188, pl. 12, figs 9, 10.

*Verruca cookei* Rosell, 1989: 299, pl. 11, figs r, s, u, v

**Table 1** Geographic coordinates, water depths, sampling details, year of sampling and post-mortem ages at time of specimen sampling of the 6 specimens of *Nautilus macromphalus* with cirripede encrustations. From Tomašových *et al*. 2016

| Station | Collection number | Latitude | Longitude | Depth (m) | Region | Sampling gear | Campaign | Year | Post-mortem age (y) |
|---|---|---|---|---|---|---|---|---|---|
| CP30 | UBGD 30739 | –23.1608 | 166.6808 | 1140 | S of New Caledonia | beam trawl | BIOCAL | 1985 | 106 |
| CP45 | UBGD 30741 | –22.789 | 167.2467 | 430 | SW of Isle of Pines | beam trawl | BIOCAL | 1985 | 358 |
| CP45 | UBGD 30735 | –22.789 | 167.2467 | 430 | SW of Isle of Pines | beam trawl | BIOCAL | 1985 | 416 |
| CP232 | UBGD 30740 | –21.5635 | 166.45117 | 760–790 | E of New Caledonia | beam trawl | BIOGEOCAL | 1987 | 227 |
| DW44 | UBGD 30748 | –22.7883 | 167.2383 | 440–450 | SW of Isle of Pines | dredge | BIOCAL | 1985 | 173 |
| DW08 | UBGD 30750 | –20.5725 | 166.8983 | 435 | NW of Lifou Island | dredge | BIOCAL | 1985 | 280 |

(non *Verruca cookei* Pilsbry, 1927).

*Verruca cookei* Rosell, 1991: 33 (non *Verruca cookei* Pilsbry, 1927).

*Metaverruca recta* Aurivillius, 1898 — Buckeridge, 1994: 116, figs 13a–f; Buckeridge, 1997: 143, fig 7b; Young, 1998: 52; Poltarukha & Zevina 2006a: 158–159, fig. 8; Poltarukha & Zevina 2006b: 174; Chan *et al.* 2009: 277–278, figs. 242–244; Chan *et al.* 2010: 36, figs 2G, 25, 26; Poltarukha 2013: 42, fig. 6; Poltarukha 2014: 32–33

**Material: UBGD 30750** – stn DW 08 (20°34′21″S, 166°53′53.88″E); depth 435 m; 1 specimen attached to the left side of the *Nautilus* shell (Fig. 2a, a2). Note: Buckeridge (1994: 116) recorded *M. recta* as a benthic species from this station.

**Description:** A large, low conic verrucid with steep sides and operculum sub-parallel to base; orifice D-shaped; exterior smooth with fine, well-spaced concentric growth lines; movable tergum and movable scutum with 3 articular ribs; rostrum and carina articulating with up to 7 ribs; caudal appendages short.

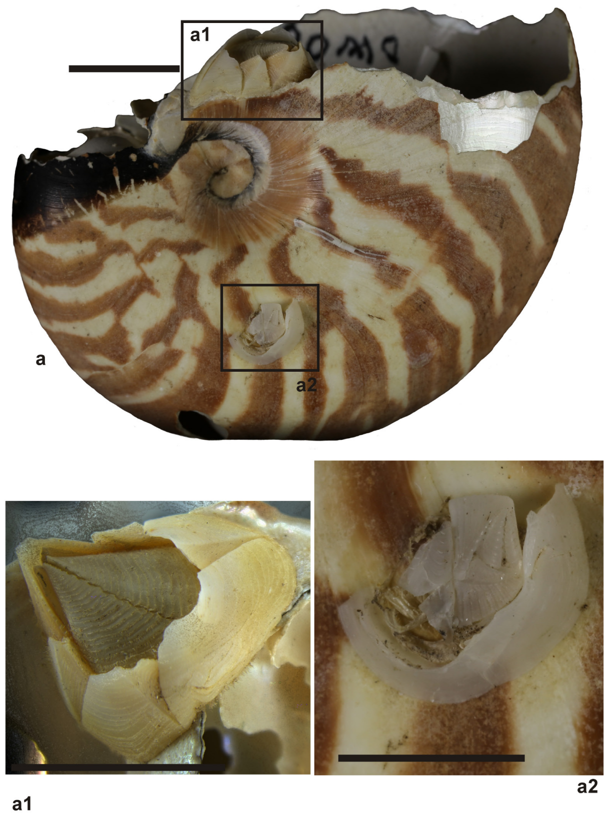

**Figure 2** (a) UBGD 30750 – *Nautilus macromphalus* with 2 attached sessile cirripedes (scale bar 10 mm); (a1) The balanomorph *Hexelasma velutinum* Hoek, 1913. Scale bar 10 mm; (a2) The verrucomorph *Metaverruca recta* (Aurivillius, 1898) (scale bar 5 mm).

**Remarks:** *M. recta* is a cosmopolitan species that is widely distributed in New Caledonian waters. It is known to live at depths ranging from 160 to 2110 m (Buckeridge 1994). This bathymetric range overlaps with the range of *N. macromphalus* in New Caledonia. There is only 1 specimen of this verrucid in the material studied; it is attached on the left flank of UBGD 30750. Although this shell, retrieved from 435 m, retains its original color stripes, the surface is affected by fine-scale dissolution and by the loss of original sheen, and no signs of periostracum are preserved. The barnacle had to settle on an already dead shell of *Nautilus*, as the minimum post-mortem age (lower 95% confidence interval is 150 years, see table 1 in Tomašových *et al*. 2017) clearly exceeds the maximum estimable life span of this verrucid specimen.

**Distribution:** Extant in seas off Japan, Philippines, South China Sea, Southwest Pacific, North West Pacific, Taiwan Island. Lower Miocene of New Zealand.

**Order Balanomorpha Pilsbry, 1916**

**Family Bathylasmatidae Newman & Ross, 1971**

**Genus *Hexelasma* Hoek, 1913**

***Hexelasma velutinum* Hoek, 1913**

(Fig. 2a, a1; Figs 3–5).

*Hexelasma velutinum* Hoek, 1913: 246; Broch, 1931: 53; Hiro, 1933: 79; Utinomi, 1968: 30; Newman & Ross, 1971: 155; Newman & Ross, 1976: 46; Foster, 1981: 356–358; Jones 2000: 273, fig. 64, tabs 28–31; Liu & Ren 2007: 328, fig. 146; Chan *et al.* 2009: 183–184, figs 155–157; Chan *et al.* 2010, p. 41–43, fig. 29–30.

**Material: UBGD 30750** – stn DW 08 (20°34′21″S, 166°53′53.88″E); depth 435 m; 1 specimen with opercula, attached inside the broken body chamber, near the umbilical area (Fig. 2a, a1); **UBGD 30735** – stn CP 45 (22°47′20.4″S, 167°14′37.32″E); depth 430 m; 1 specimen (without opercula) attached on the ventral side and 1 hexelasmatine base (Fig. 4); Calcareous bases preserved on two other specimens may also belong to this species: **UBGD 30741** – stn CP 45 (22°47′20.4″S, 167°14′37.32″E); depth 430 m; two ?hexelasmatine bases (Fig. 5b, b1); **UBGD 30748** – stn DW 44/2 (22°47′17.88″S, 167°14′17.88″E); depth 440-450 m; one ?hexelasmatine base (Fig. 5).

**Description:** Shell high conic, comprising 6 wall plates: rostrum (R), carina (C) and paired first carino-latera ($CL^1$) and second carino-latera ($CL^2$); alae present on C, $CL^1$ and $CL^2$; radii absent, lower 2/3 of alar growth lines parallel to margins of adjacent plate, superior margin of alae with fine welting. Exterior cream-white due to cover by a thin straw-colored chitinous integument, compartments and opercula with parallel growth lines delineated by fine, short setae; interior surface of compartments with thin, yellow-brown, longitudinal chitinous stringers. Compartments inflected inwards basally, basis very thin, calcareous [some writers (e.g. Chan 2009) state, in error, that the basis is membranous]. Scutum triangular with slightly convex articulating and occludent margins, articular ridge low, adductor muscle pit central, but very weak; tergum with broadly convex carinal margin, articulating margin and basal margins slightly concave, spur extending shortly beyond basi-scutal angle and removed from basi-scutal angle by slightly less than its own width, ridges for lateral depressor muscles well formed; exterior with shallow tergal furrow.

**Note:** The vertical chitinous stringers that occur within the shell wall of *Hexelasma velutinum* are degraded (and lost) when the barnacle dies and disarticulates. Depending on depth, microbial activity and temperature, degradation may be well advanced in several months. In some instances, a small part of the shell wall may re-

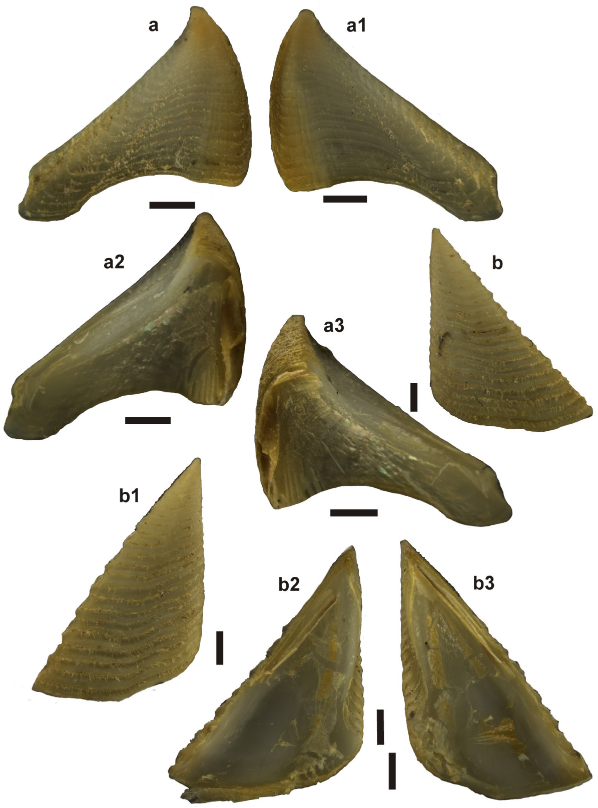

**Figure 3** UBGD 30750 – Opercular plates of *Hexelasma velutinum* Hoek, 1913. (a–a1) External view of terga; (a2–a3) internal view of terga; (b–b1) external view of scuta; (b2–b3) internal view of scuta (scale bars 1 mm).

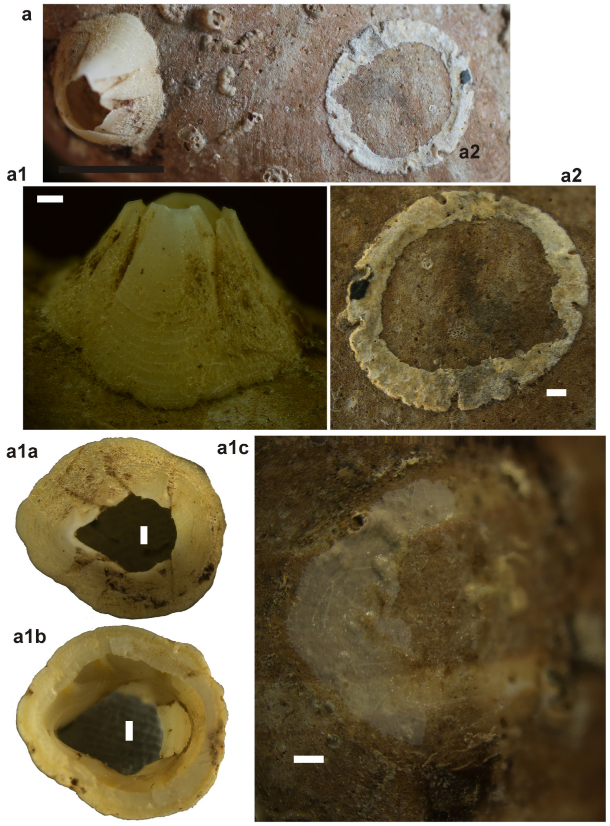

**Figure 4** UBGD 30735 – *Nautilus macromphalus* with *Hexelasma velutinum* Hoek, 1913. (a) Top view of operculum of *H. velutinum*, showing base of *H. velutinum* as the second epibiont, attached over an agglutinated foraminifer (scale bar 10 mm); (a1) carinal view of *H. velutinum* (scale bar 1 mm); (a2) base of *H. velutinum* (scale bar 1 mm); (a1a) opercular view of *H. velutinum*, (scale bar 1 mm). (a1b) View of base (scale bar 1 mm); (a1c) view of the cirripede base overgrowing agglutinated foraminifers (the primary epibiont) (scale bar 1 mm).

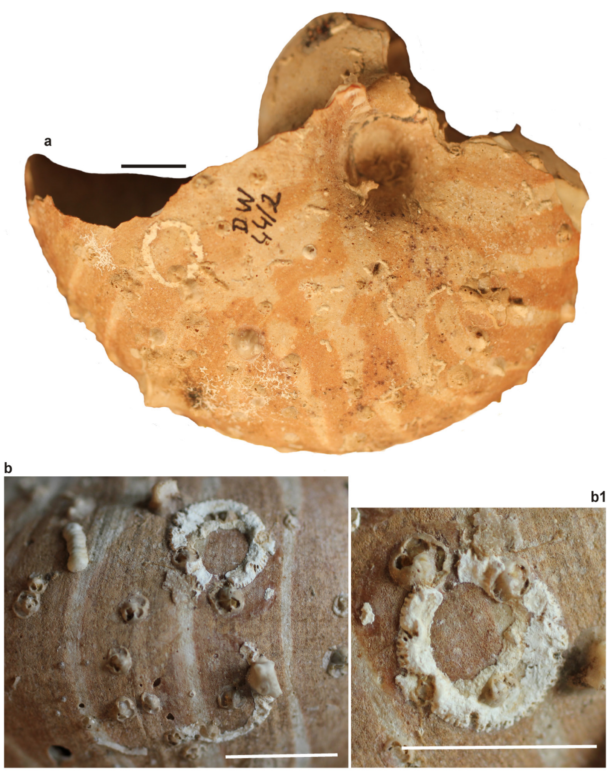

**Figure 5** (a) UBGD 30748 – *Nautilus macromphalus* with a balanomorph base (scale bar 1cm) – ?*Hexelasma velutinum* Hoek, 1913. (b) UBGD 30741 – *N. macromphalus* with the remains of 2 non-porous calcareous bases, likely belonging to *H. velutinum* on the ventral surface of the *Nautilus* shell. (b1) Detailed view, showing remnants of internal longitudinal ribbing and small cavities (where there had been chitinous stringers within the compartmental wall) at the edge of the calcareous base (scale bars 1 cm).

main attached to the base. At the base-wall abutment zone, small cavities can be seen where the chitin once existed (Fig. 5b–b1), and these look superficially like basal pores (i.e. openings to a porous calcareous base, as with *Amphibalanus*).

**Distribution:** Japan, Philippines, South China Sea, Southwest Pacific. A depth range of 204–560 m was provided by Hoek (1913: 246). However, Hoek confused 2 taxa in his description; see Foster (1979: 79). Pilsbry (1916: 330) and Newman and Ross (1976: 46) both give a depth range of 204–390 m, so the studied specimens (430–450 m) may be one of the deepest records for this species. This species does not have any fossil record.

## Systematic ichnology

**General remarks:** Barnacles quickly lose soft tissue and opercula after their death. The compartments may detach from the base, which can be either calcareous or membranous. Two types of cirripede attachment were observed on these *Nautilus* shells. The first type is represented by calcareous, non-porous, very thin bases typical of hexelasmatines, such as *H. velutinum* (Fig. 4a1c). The second type is represented by etching scars typical of cirripedes that lack a mineralized base, such as with the Verrucomorpha and most of the Chthamalidae. This type of attachment can corrode the substrate encrusted by the barnacle (Buckeridge & Newman 2017), depending on the degree of substrate preservation and its strength. These unmineralized scars contain significantly less information about the identity of the producer. Therefore, we assign them to a new ichnospecies on the basis of their distinctive preservation and shape (Boekshoten 1967; Miller & Brown 1979; Mayoral 1986; Bromley & Martinell 1991; Santos *et al*. 2005).

Gale (2018, and references therein) discussed host selectivity of barnacles, especially during the Mesozoic. The specimens that may have colonized nautiloids *in vivo* are too few in number to confirm any selectivity.

**Ichnogenus** *Anellusichnus* Santos *et al.* 2005

*Anellusichnus ellipticus* isp. nov.

(Figs 6–9)

"Verrucomorph bioerosion" – Buckeridge & Newman 2017: 230, fig. 4.

**Diagnosis:** Circular or subcircular patterns defined by a shallow, non-crenulate, exterior ellipsoidal furrow; inside this, the structure exhibits fine, eccentric etched rings with no central depression.

**Material: Holotype UBGD 30740** – stn CP 232 (21°33′46.8"S, 166°27′4.32"E); depth 760–790 m; large attachment scar marked as "H" in Figure 6; **Paratype series UBGD 30740** – stn CP 232 (21°33′46.8"S, 166°27′4.32"E); depth 760–790 m; 71 completely preserved attachment scars and 14 fragments (due to shell breakage during or after the sampling); **UBGD 30739** – stn CP 30 (23°09′38.88"S, 166°40′50.88"E); depth 1140 m; 1 attachment scar (Fig. 7).

**Description:** Circular and irregularly elliptical attachment scars ranging from 3.0 to 9.4 mm in diameter that represent distinct growth phases. These scars may overlap, indicating that the producer colonized the shells of *Nautilus* in multiple phases (see Fig. 6a2–a3). Four types of scar preservation can be distinguished. First, if the barnacle is attached to a relatively fresh shell surface, it will leave an almost invisible circular trace without significant discoloration of the substrate, which can, nonetheless, be seen under SEM (Fig. 8). Second, some traces are associated with discoloration, leaving circular or elliptical traces, but with no sign of bioerosion (Fig. 9a,c–e). Third, some scars (still associated with loss of color pigments) display a marginal ring (produced by etching). The center of a trace series is, thus, elevated

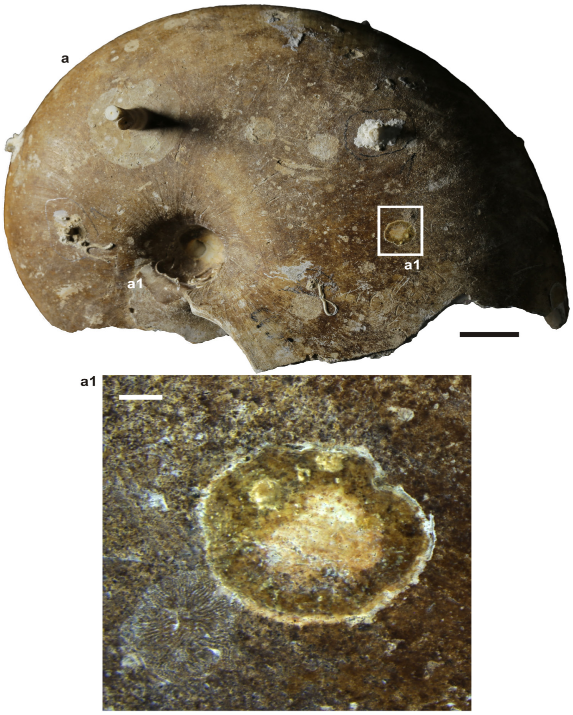

**Figure 7** (a) UBGD 30739 – *Nautilus macromphalus* with a cirripede attachment scar (scale bar 10 mm). (a1) Detailed view of the attachment scar (scale bar 1 mm), tentatively assigned to *Anellusichnus ellipticus* isp. nov., probably produced by a verrucid post-mortem the Nautilus. The green discoloration is likely due to algal growth.

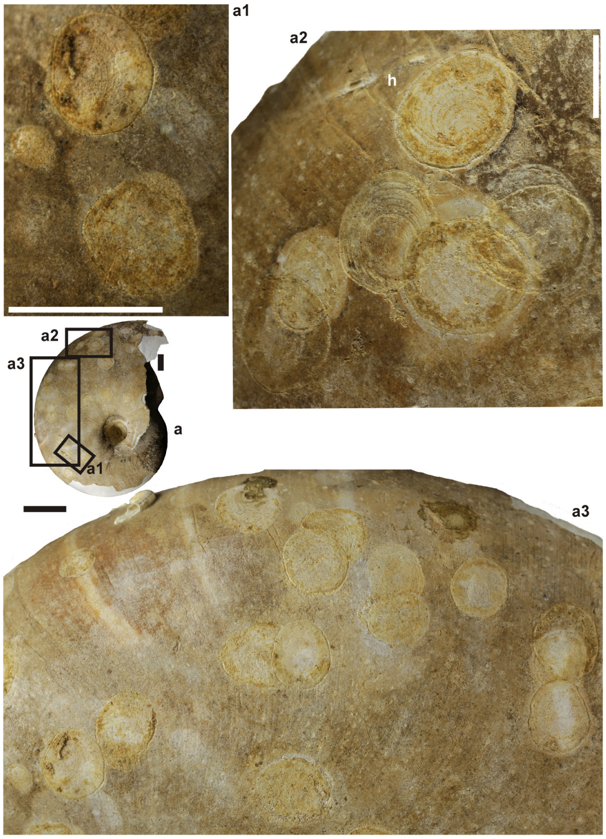

**Figure 6** (a) UBGD 30740 – *Nautilus macromphalus* with verrucomorph attachment scars (scale bar 10 mm) belonging to ichnospecies *Anellusichnus ellipiticus* isp. nov. (a1) Detailed view of corroded attachment scars showing up to 8 concentric rings (scale bar 5 mm); (a2) detailed view of 9 verrucomorph attachment scars; the one in the middle shows 18 internal rings (scale bar 5 mm). (a3) Detailed view of 17 verrucomorph attachment scars (scale bar 10 mm)

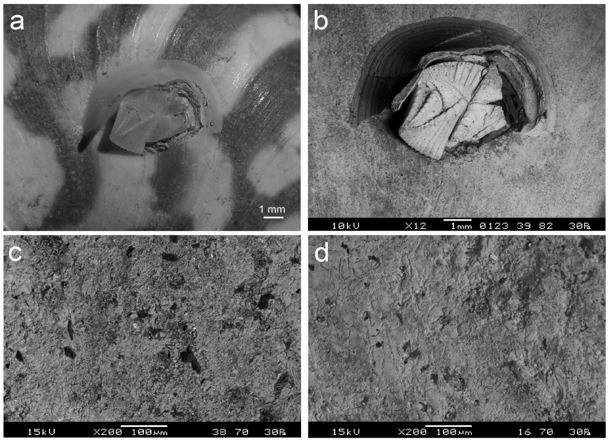

**Figure 8** (a–b) The upper row shows the specimen of *Metaverruca recta* on UBGD 30750 that has not left any distinct dissolution scar or furrow; only a very indistinctive circular scar can be distinguished on the SEM photo. (c–d) The lower row shows that the external surface within the attachment trace is affected by microbioerosion and fine-scale alteration of the prismatic layer (c). The external preservation outside of the trace is also affected by high frequency of bioerosion (d).

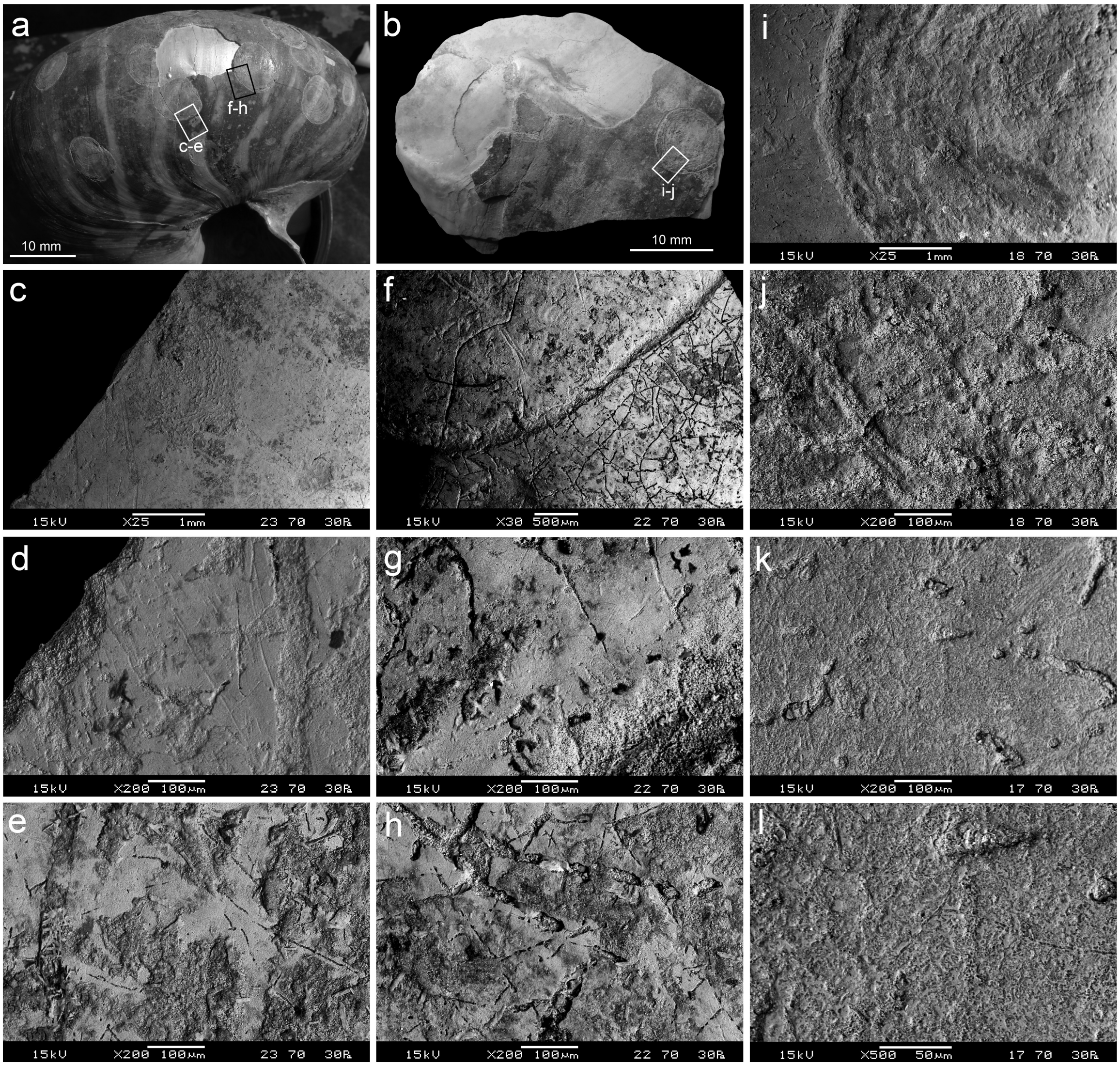

**Figure 9** Three types of verrucomorph scar appearance and preservation in the specimen UBGD 30740. (a) General view of the lateral–ventral part of the left side of the specimen. (b) Broken fragment of the body chamber from the more altered right side of the specimen. Left column (c–e): Attachment trace preserved on the left, better-preserved flank (with color preservation) does not leave any clear trace of dissolution (white dots outline the trace). The trace is, however, associated with discoloration (loss of reddish pigment). The surface outside of the trace is affected by bioerosion (e), similarly as is the surface within the trace (d). The middle column (f–h): The attachment trace on the left flank, associated with discoloration (loss of reddish pigment) shows just a marginal concentric dissolution ring (f). The center of the trace is not affected by dissolution (g). Right column (i–l): The trace with distinct eccentric rings on the altered right flank. The surface inside the trace is very irregular (i) due to the etching by the barnacle (j). The surface outside of the trace (k–l) is significantly microbioeroded, which can be nicely seen under great magnification (the shell is completely perforated with microborings, thus producing a very porous fabric).

and does not show any dissolution (Fig. 9a,f–h). Fourth, some scars show multiple eccentric rings enhanced by dissolution (Fig. 9b,i–l). Up to 18 eccentric rings (i.e. growth lines) can be distinguished within the scars in the better-preserved specimens (Fig. 6a2). There is no central depression. The external furrow is developed in large specimens only, and this represents a stasis in base expansion.

**Interpretation:** Buckeridge & Newman (2017) discussed bioerosion caused by the verrucid cirripede *Verruca nuciformis* Buckeridge, 1983. In their material, the substrate is also aragonitic (gastropods), although it shows greater decortication than seen in any of the *Nautilus* shells studied here. Importantly, numerous specimens of *V. nuciformis* remained attached to the substrate. After removal of several specimens of *V. nuciformis* from gastropod shells, ellipsoidal bioerosion patterns that conform to *Anellusichnus ellipticus* isp. nov. were exposed (Fig. 10).

Therefore, we conclude that *A. ellipticus* isp. nov. is produced by a verrucomorph barnacle. Buckeridge (1997) records 11 species of verrucomorph barnacle from New Caledonia waters, but only 1 species is particularly common, *Metaverruca recta*, and, importantly, this species has a non-sinusoidal compartment wall. As only 1 living verrucomorph (*M. recta*) was found on any of these *Nautilus* shells, and as this species is common in the region, it is parsimonious to assume that *M. recta* is the species most likely to have formed the scars. However, we note that any verrucomorph that has a non-sinusoid outline could be the producer, including the significantly rarer *Metaverruca pacifica* Buckeridge, 1994 and *Metaverruca macani* (Stubbings, 1936).

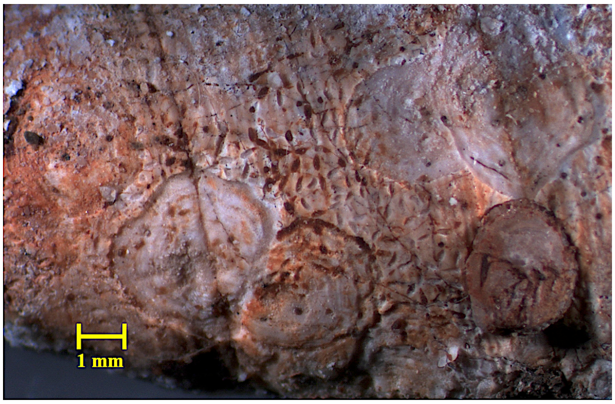

**Figure 10** Post-mortem bioerosion of mollusc shells by *Verruca nuciformis* Buckeridge, 1983 (Early Miocene, New Zealand). Bioerosion arises through dissolution of the host surface of the mollusc shell (*Tropicolpus* sp.) by the barnacle. The *Tropicolpus* sp. is also heavily bored by acrothoracicans, and this is most evident on what appears to have been a surface above the sediment. Unlike the mollusc shell, the verrucid shells that are attached to the shell are not decorticated (image from Buckeridge & Newman 2017: 230). This reflects a period of time between death of the mollusc and encrustation by the verrucid, during which the surface of the aragonitic mollusc shell was lost.

**Remarks:** The absence of a central depression in these scars distinguishes *A. ellipticus* isp. nov. from *Centrichnus concentricus* Bromley & Martinell, 1991. However, *A. ellipticus* isp. nov. also differs from *Anellusichnus circularis* Santos *et al*. 2005 by having almost completely flat relief and numerous eccentric internal rings. A non-sinusoidal outer margin distinguishes it from the *Anellusichnus undulatus* Santos *et al*. 2005.

**Distribution:** *Metaverruca recta* presently occurs in the Western Pacific and appeared for the first time in the Lower Miocene.

**Etymology:** Morphologic. From the Latin *ellipticis* (= oval).

## DISCUSSION

### *Nautilus* habitat and distribution

*Nautilus* shells provide potential substrates for the attachment of a diverse group of encrusting organisms. An understanding of living activities of *Nautilus* is formative when assessing whether encrustation occurred while the animals were alive or post-mortem during their floating stage and/or on the sediment–water interface in shallow-water environments (where they can be transported) or bathyal and deep-water environments. Following field observations, Barord (2015) argued that *Nautilus* is an obligate scavenger, feeding primarily on benthos. As *Nautilus* is vulnerable to predators, it is generally not found in the open water column, especially during daylight (Dunstan *et al*. 2011); rather, it lives near its food source, in close association with reef slopes and with the sea bed (Ward & Martin 1980; Dunstan *et al*. 2011).

*Nautilus macromphalus* lives in waters of the southwestern Pacific Ocean, especially near New Caledonia and Loyalty Islands, where it is generally found at water depths between 300 and 400 m (Saunders & Landman 1987). They are less frequent at shallow depths (depending on season) and their maximum depth does not exceed 700 m (Dunstan *et al*. 2011) as hydrostatic pressure can cause shells to implode (Saunders & Ward 2010). Those nautiluses that venture into deeper waters and survive require time at shallower depths to regain neutral buoyancy. An equilibration period generally takes place on substrates at depths of approximately 200 m (Dunstan *et al.* 2011), and this period of low activity may provide some opportunities for colonization of living *Nautilus* shells by bottom-dwelling encrusting organisms with low dispersal abilities.

## Identification of scar producers

Some specimens on *N. macromphalus* bear circular attachment scars without any shelly or body remains and these are here interpreted as being of cirripede origin (in particular, verrucomorph barnacles). We are aware that similar scars may be produced by other organisms. For example, Landman *et al*. (1996: 548) assigned sub-circular pits on ammonite shells to limpets. However, these limpet "home scars" are shallow depressions and, thus, morphologically differ from the barnacle attachment scars observed here. The nature of barnacle attachment scars, including those made by verrucomorphs, is relatively well known, based on studies of the well-preserved material (Bromley & Martinell 1991; Santos *et al*. 2005; Buckeridge & Newman 2017).

## The role of periostracum in deterring epibionts

The settlement of epibionts on living *Nautilus macromphalus* tends to be highest near or in the umbilicus, where the smooth, organic periostracum that covers most of the shell is absent (Hamada 1983). Interestingly, *Nautilus* shells with barnacle epibionts described by Hamada (1983) were damaged, and barnacles were attached to "rugae" of the repaired shell (Hamada 1983). It was thus hypothesized that periostracum may inhibit the colonization of boring and encrusting epibionts on shells of *Nautilus* (Seuss *et al*. 2015). However, the periostracum in *N. macromphalus* is extremely thin (2 µm according to Ward 1987). Although 5 of 24 shells had remains of original sheen preserved, and 9 shells had some remnants of original red stripes (Tomašových *et al*. 2017), no traces of original periostracum can be observed on the dead shells in this collection (Figs 8–9). Therefore, the original periostracum probably degrades very quickly, and is thus, not limiting colonization by encrusting organisms and/or their boring activities soon after death. Given that all shells of *Nautilus* analyzed here persisted in the taphonomic active zone (close to sediment-water interface) for at least a century, this deterrent mechanism seems not to have affected barnacle colonization. Taphonomic patterns on *Nautilu*s shells from New Caledonia bathyal environments indicate that some shells were oriented in a relatively stable position, with rare overturning, for decades, providing ideal substrates for colonization by epibionts that require stable substrate (Tomašových *et al*. 2017).

## Preservation of verrucomorph traces

Two processes are associated with the attachment of verrucomorph barnacles, including discoloration and dissolution. Discoloration precedes any signs of dissolution in some scars (Fig. 9a), and is probably induced by pigment leaching in the uppermost parts of the prismatic layer. The degree of bioerosion and discoloration induced by verrucomorph barnacles varies as a function of surface preservation of *Nautilus* shells. Traces on fresh, unaltered surfaces of UBGD 30750 leave no scars (and are not associated with discoloration, Fig. 8) and traces observed on the left flank of UBGD 30740 show either no signs of dissolution (Fig. 9a,c) or just a marginal furrow produced by dissolution (Fig. 9a,f). In contrast, traces on degraded surfaces on the right side of the UBGD 30740 produce deeper ellipsoidal traces associated with discoloration (Fig. 9b,i). As noted, similar traces were observed on gastropod shells (Fig. 10) by Buckeridge and Newman (2017). Small-scale bioerosion occurring outside of scars is more intense than within the scars, indicating that the bioerosion occurred in parallel with encrustation.

## The duration of floating stage in *Nautilus*

Chamberlain *et al*. (1981) showed that shells of *Nautilus* at 300 m or more will not ascend to surface waters because high ambient hydrostatic pressure at larger depths generates a high rate of water influx into the phragmocone. Therefore, unless they are already encrusted, they will not acquire shallow-water pelagic cirripedes such as *Lepas* and *Conchoderma*. *Nautilus* that die at shallower depths (i.e. 5−150 m) may drift in surface waters for some time before buoyancy is lost, at which point they sink to the seabed. House (1987) and Seuss *et al.* (2015) note that post-mortem, shells may retain buoyancy for months before settling to the bottom; over this period, there is potential for them to have drifted for many hundreds of kilometers. Final resting sites of dead shells of *Nautilus* that have been recorded off New Caledonia range from the upper photic (1–50 m) to the mesobathyal zone (Mapes *et al*. 2010a; Seuss *et al*. 2015) up to 1140 m (Tomašových *et al*. 2017). However, all specimens of *N. macromphalus* analyzed here occur within the geographic and for the most part also bathymetric range of living populations (Tomašových *et al*. 2016). Therefore, these shells most likely did not float for long. In New Caledonia, a subset of dead shells is transported to beach and shallow–subtidal environments (Mapes *et al*. 2010a,b), but shells preserved on slopes most likely represent shells that did not drift far

(i.e. the *Nautilus* died at greater depths). Therefore, the phase when floating shells were exposed to settlement by planktic larvae in the upper parts of the water column was probably short. It is possible that in slope environments with steep topographic gradients, even subtle sedimentary transport can lead to redeposition of shells colonized by shallow-water epizoic communities to deeper habitats. However, this scenario is not observed (i.e. all barnacles are typical of deep-water habitats). As noted, lepadids frequently colonize *Nautilus* from the Philippines and Papua New Guinea (see Landman *et al*. 1996 for an overview) and their attachment has been observed also in long-term rearing aquarium experiments (Hamada 1983). Although there is no evidence for the lepadids on any of our shells, they do not leave any attachment scars, and, thus, their colonization history cannot be inferred on the basis of empty shells. Tomašových *et al*. (2017) showed that the percentage of encrusters initially increased with post-mortem age in *N. macromphalus* but then declined in the oldest shells where there was a high degree of dissolution and decortication of external shell layers.

### Benthic encrustation in bathyal environments on the sediment–water interface

On UBGD 30750, the specimen of *M. recta* possesses articulated opercula and shows some traces of organic tissue (on a shell that had been on the sea floor for at least 150 years); furthermore, the specimen of *H. velutinum* is attached to internal surfaces of the shell.

In UBGD 30740, higher density of attachment scars of *A. ellipticus* isp. nov. on one of the flanks, coinciding with higher degree of shell alteration (with dark, goethite-rich coatings), strongly indicates that the barnacle overgrowth occurred on the upper exposed flank surface as a consequence of post–mortem colonization (e.g. Schmid-Röhl & Röhl 2003). All shells collected here are most likely to have been colonized post-mortem. The exception could be some specimens belonging to *Hexelasma*, preserved only as calcareous eroded bases. These bases can be rather old and may have settled on the *Nautilus* when still alive. Seuss *et al*. (2015) suggested that *Nautilus* shells preserved in backshore environments were initially deposited in subtidal environments where they were exposed to borers and encrusters. In contrast, shells preserved in bathyal environments do not seem to possess such complex history in terms of their barnacle encrustation. The periostracum is lost due to erosion and microbial activity very quickly, exposing the aragonitic shell for encrusters and borers. If dead shells are altered to some degree and colonized by verrucomorphs, attachment scars form (e.g. *A. ellipticus* isp. nov.). Rolling and partial embedding of the shell in the sediment may kill earlier settlements of cirripedes, which, in turn, are preserved only as calcareous bases or attachment scars.

## CONCLUSION

The history of epifaunal colonization on cephalopod shells has useful applications in unravelling the dispersal and post-mortem fates of shells in the fossil record and can be informative about the length of residence time on the sea floor. Encrusters can colonize cephalopod shells during their life or post-mortem and discriminating between these two modes can be difficult in the fossil record if only external surfaces are encrusted. Analysis of these shells of *Nautilus* shows that epibionts primarily colonized empty shells on the seafloor rather than during their floating stage. If colonization occurs on relatively fresh shells, attachment of verrucomorph barnacles leaves no clear discoloration or dissolution scars. Colonization of worn shells by verrucomorph barnacles leaves distinct attachment scars and, thus, has significant potential to assist in interpretation of the fossil record.

## ACKNOWLEDGMENTS

We thank D. Marchand for introducing us to specimens collected by Biocal and Biogeocal cruises, and J. Thomas for access to specimens deposited at the Université de Bourgogne. We also thank Jan Sklenář (National Museum Prague) for free access to a light microscope and Canon EOS 6D camera, and Martin Mazuch from the Charles University in Prague for the SEM photos. TKs research was supported by the Ministry of Culture of the Czech Republic (IP DKRVO 2019/2.III. a, National Museum, 00023272) and MKVs research was supported by the Center for Geosphere Dynamics (UNCE/SCI/006) and by the RVO 67985831 of the Institute of Geology of the Czech Academy of Sciences. JS and AT were funded by the Slovak Scientific Grant Agency (VEGA 2/0169/19) and by the Slovak Agency for Research and Development (APVV 17-0555).

**Cite this article as:**

## Cite this article as:

Buckeridge J, Kočí T, Schlögl J, Tomašvých A, Kočová Veselská M (2019). Deep‐water cirripedes colonizing dead shells of the cephalopod *Nautilus macromphalus* from New Caledonian waters. *Integrative Zoology* **14**, 561–75.

## References

[inz212389-bib-0001] Aurivillius CWS (1892). Neue Cirripeden aus dem Atlantischen, Indischen und Stillen Ocean. Oefverigt af K. Vetenskapsakdemiens Förhandlingar 3, 123–34.

[inz212389-bib-0002] Aurivillius CWS (1894). Studien über Cirripeden. Kongliga Svenska Vetenskaps Academiens Handlingar Uppsala 26, 5–107.

[inz212389-bib-0003] Baird GC , Brett CE , Frey RC (1989). Hitchhiking epizoans on orthoconic cephalopods: Preliminary review of the evidence and its implications. Senckenbergiana lethaea 69, 439–65.

[inz212389-bib-0004] Barord GJ (2015). On the biology, behaviour and conservation of the chambered Nautilus, Nautilus sp. (doctoral thesis). The City University of New York/Proquest, New York.

[inz212389-bib-0005] Boekschoten GJ (1967). Palaeoecology of some Mollusca from the Tielrode sands (Pliocene, Belgium). Palaeogeography, Palaeoclimatology, Palaeoecology 3, 311–62.

[inz212389-bib-0006] Broch H (1931). Papers from Dr. Th. Mortensen's Pacific Expedition 1914–1916, LVI. Indomalayan Cirripedia. Videnskabelige Meddelelser fra Dansk Naturhistorisk Forening i Kobenhavn 91, 1–146.

[inz212389-bib-0007] Bromley RG , Martinell J (1991). *Centrichnus*, new ichnogenus for centrically patterned attachment scars on skeletal substrates. Bulletin of the Geological Society of Denmark 38, 243–52.

[inz212389-bib-0008] Buckeridge JS (1994). Cirripedia Thoracica: Verrucomorpha of New Caledonia, Indonesia, Wallis and Futuna Islands. In: Crosnier A, ed. Resultats des Campagnes MUSORSTOM, 12, Memoires du Museum national d'Histoire naturelle 4, 87–126.

[inz212389-bib-0009] Buckeridge JS (1997). Cirripedia Thoracica: New Ranges and Species of Verrucomorpha from the Indian and Southwest Pacific Ocean, Résultats des campagnes MUSORSTOM, 18, Mémoires du Muséum national d'histoire naturelle 176, 125–49.

[inz212389-bib-0010] Buckeridge JS , Newman WA (2017). The “Tears of the Virgin” at Lakes Entrance, Victoria were made by the intertidal barnacle *Chthamalus antennatus* Darwin (Cirripedia: Thoracica). Integrative Zoology 12, 228–36. 27782367 10.1111/1749-4877.12244

[inz212389-bib-0011] Chamberlain JA , Ward PD , Weaver JS (1981). Post-mortem ascent of *Nautilus* shells: implications for cephalopod paleobiogeography. Paleobiology 7, 494–509.

[inz212389-bib-0012] Chamberlain JA (2010). Locomotion of *Nautilus* In: Saunders WB, Landman NH, eds. Nautilus. The Biology and Paleobiology of a Living Fossil. Topics in Geobiology, Vol. 6, pp. 489–525.

[inz212389-bib-0013] Chan BKK (2009). Shallow water and deep‐sea barnacles (Crustacea: Cirripedia: Thoracica) collected during the Philippine Panglao 2005 expedition, with description of two new species. Raffles Bulletin of Zoology 20, 47–82.

[inz212389-bib-0014] Chan BKK , Prabowo RE , Lee K-S (2009). Crustacean Fauna of Taiwan: Barnacles, volume 1 – Cirripedia: Thoracica Excluding the Pyrgomatidae and Acastinae. pp. 1–298. National Taiwan Ocean University, Keelung.

[inz212389-bib-0015] Chan BKK , Prabowo RE , Lee K-S (2010). North West pacific deep‐sea barnacles (Cirripedia, Thoracica) collected by the Taiwan expeditions, with descriptions of two new species. Zootaxa 2405, 1–47.

[inz212389-bib-0016] Davis RA , Mapes RH , Klofak SM (1999). Epizoa on externally shelled cephalopods. In: Rozanov AY, Shevyrev AA, eds. Fossil Cephalopods: Recent Advances in Their Study. Russian Academy of Sciences Paleontological Institute, Moscow, pp. 32–51.

[inz212389-bib-0017] Dennison KJ , Keiser JA , Buckeridge JS , Bishop PJ (2004). *Post mortem* cohabitation – Shell growth as a measure of elapsed time: A case report. Forensic Science International 139, 249–54. 15040925 10.1016/j.forsciint.2003.10.015

[inz212389-bib-0018] Donovan SK (1989). Taphonomic significance of the encrustation of the dead shell of recent *Spirula spirula* (Linné) (Cephalopoda: Coleoidea) by *Lepas anatifera* Linné (Cirripedia: Thoracia). Journal of Paleontology 63, 698–702.

[inz212389-bib-0019] Dunstan A , Ward P , Marshall NJ (2011). Vertical distribution and migration patterns of Nautilus pompilius. PLoS ONE 6, e16311. 21364981 10.1371/journal.pone.0016311 PMC3043052

[inz212389-bib-0020] Foster BA (1979). The marine fauna of New Zealand: Barnacles (Cirripedia: Thoracica), Memoirs of the New Zealand Oceanographic Institute 69, 1–160.

[inz212389-bib-0021] Foster BA (1981). Cirripedes from ocean ridges north of New Zealand. New Zealand Journal of Zoology 8, 349–67.

[inz212389-bib-0022] Gale A (2018). Stalked barnacles (Cirripedia, Thoracica) from the Upper Jurassic (Tithonian) Kimmeridge Clay of Dorset, UK; paleoecology and bearing on the evolution of living forms. Proceedings of the Geologists' Association. Available from URL: 10.1016/j.pgeola.2018.01.005.

[inz212389-bib-0023] Hamada T (1983). Preliminary report on same nautilus drifts and the epifauna on *Nautilus* shells in a living state from the Tañon Strait, the Philippines. Kagoshima University Research Center for the South Pacific, Occasional Papers 1, 36–9.

[inz212389-bib-0024] Hautmann M , Ware D , Bucher H (2017). Geologically oldest oysters were epizoans on Early Triassic ammonoids. Journal of Molluscan Studies 83, 253–60.

[inz212389-bib-0025] Hiro F (1933). Report on the Cirripedia collected by the surveying ships of the Imperial Fisheries Experimental Station on the continental shelf bordering Japan. Records of the Oceanographic Works in Japan 5, 11–84.

[inz212389-bib-0026] Hoek PPC (1913). The Cirripedia of the Siboga Expedition B. Cirripedia Sessilia. Siboga-Expeditie 31b, 129–275.

[inz212389-bib-0027] House MR (1987). Geographic distribution of *Nautilus* shells. In: Saunders WB, Landman NH, eds. Nautilus: The Biology and Paleobiology of a Living Fossil. Plenum Press, New York pp. 53–64.

[inz212389-bib-0028] Jones DS (2000). Crustacea Cirripedia Thoracica: Chionelasmatoidea and Pachylasmatoidea (Balanomorpha) of New Caledonia, Vanuatu and Wallis and Futuna Islands, with a review of all currently assigned taxa. Mémoires du Muséum national d'histoire naturelle 184, 141–283.

[inz212389-bib-0029] Jones DS (2001). The Cirripedia of New Caledonia. Compendium of Marine Species from New Caledonia. Institut de Recherche pour le Développement, Nouméa, pp. 289–94.

[inz212389-bib-0030] Keupp H , Röper M , Seilacher A (1999) Paläobiologische Aspekte von syn vivo‐besiedelten Ammonoideen im Plattenkalk des Ober‐Kimmeridgiums von Brunn in Ostbayern. Berliner Geowissenschaftliche Abhandlungen E 30, 121–45.

[inz212389-bib-0031] Klug C , Korn D (2001). Epizoa and post‐mortem epicoles on cephalopod shells–Devonian and Carboniferous examples from Morocco. Berliner geowissenschaftliche Abhandlungen E 36, 145–55.

[inz212389-bib-0032] Landman NH (1983). Barnacle attachment on live *Nautilus*: Implications for *Nautilus* growth rate. Veliger 26, 124–7.

[inz212389-bib-0033] Landman NH, Tanabe K, Davis RA (Eds) (1996). Ammoniod Paleobiology. Plenum Press, New York, 857 pp.

[inz212389-bib-0034] Landman NH , Saunders WB , Winston JE , Harries PJ (2010). Incidence and kinds of epizoans on the shells of live *Nautilus* . In: Nautilus. Springer, Dordrecht, pp. 163–77.

[inz212389-bib-0035] Landman NH , Mapes RH , Cochran JK *et al* (2014). An unusual occurrence of *Nautilus macromphalus* in a cenote in the Loyalty Islands (New Caledonia). PLoS ONE 9, e113372. 25470257 10.1371/journal.pone.0113372 PMC4254454

[inz212389-bib-0036] Lescinsky HL (1993). Taphonomy and paleoecology of epibionts on the scallops *Chlamys hastata* (Sowerby 1843) and *Chlamys rubida* (Hinds 1845). Palaios, pp. 267–77.

[inz212389-bib-0037] Liu RY , Ren XQ (2007). Fauna Sinica Invertebrata, Vol. 42, Crustacea Cirripedia Thoracica. Science Press, Beijing, China, 633 pp.

[inz212389-bib-0038] Luci L , Cichowolski M (2014). Encrustation in nautilids: A case study in the Cretaceous species *Cymatoceras perstriatum*, Neuquén Basin, Argentina. Palaios 29, 101–20.

[inz212389-bib-0039] Lukeneder A (2008). The ecological significance of solitary coral and bivalve epibionts on lower cretaceous (Valangianian–Aptian) ammonoids from the Italian Dolomites. Acta Geologica Polonica 58, 425–36.

[inz212389-bib-0040] Maeda H , Seilacher A (1996). Ammonoid taphonomy. In: Landman NH, Tanabe K, Davis R, eds. Ammonoid Paleobiology. Plenum Press, New York, pp. 543–78.

[inz212389-bib-0041] Mapes RH , Hembree DI , Rasor BA *et al* (2010a). Modern *Nautilus* (Cephalopoda) taphonomy in a subtidal to backshore environment, Lifou (Loyalty Islands). Palaios 25, 656–70.

[inz212389-bib-0042] Mapes RH , Landman NH , Cochran K *et al* (2010b). Early taphonomy and significance of naturally submerged *Nautilus* shells from the New Caledonia region. Palaios 25, 597–610.

[inz212389-bib-0043] Mayoral E (1986). Tafonomía y paleoecología del Plioceno de Huelva‐Bonares. Sevilla, Universidad de Sevilla, 599 pp.

[inz212389-bib-0044] Meenakshi VR , Martin AW , Wilbur KM (1974). Shell repair in Nautilus macromphalus. Marine Biology 27, 27–35.

[inz212389-bib-0045] Miller W , Brown NA (1979). The attachment scars of fossil balanids. Journal of Paleontology 53, 208–10.

[inz212389-bib-0046] Mironenko AA (2015). A new type of shell malformation caused by epizoans in Late Jurassic ammonites from Central Russia. Acta Palaeontologica Polonica 61, 645–60.

[inz212389-bib-0047] Nebelsick JH , Schmid B , Stachowitsch M (1997). The encrustation of fossil and recent sea‐urchin tests: ecological and taphonomic significance. Lethaia 30, 271–84.

[inz212389-bib-0048] Newman WA , Hessler RR (1989). A new abyssal hydrothermal verrucomorphan (Cirripedia: Sessilia): The most primitive living sessile barnacle. Transactions of the San Diego Society of Natural History 21, 259–73

[inz212389-bib-0049] Newman WA , Ross A (1971). Antarctic Cirripedia. Antarctic Research Series 14, 1–257. American Geophysical Union, Washington, DC.

[inz212389-bib-0050] Newman WA , Ross A (1976). Revision of the balanomorph barnacles; including a catalog of the species. Memoirs of the San Diego Society of Natural History 9, 1–108.

[inz212389-bib-0051] Pilsbry HA (1916). The sessile barnacles (Cirripedia) contained in the collections of the U.S. National Museum; including a monograph of the American species. Bulletin of the United States National Museum 93, 1–366.

[inz212389-bib-0052] Pitombo FB (2004). Phylogenetic analysis of the Balanidae (Cirripedia, Balanomorpha). Zoologica Scripta 33, 261–76.

[inz212389-bib-0053] Poltarukha OP (2013). Barnacles (Cirripedia: Thoracica) from the equatorial east Pacific. Russian Journal of Marine Biology 39, 51–7.

[inz212389-bib-0054] Poltarukha OP (2014). On the deep‐sea barnacle (Cirripedia, Thoracica) fauna of the southern Pacific Ocean. Byulleten' Moskovskogo Obshchestva Ispytatelei Prirody Otdel Biologicheskii 119, 28–35.

[inz212389-bib-0055] Poltarukha OP , Zevina GB (2006a). Barnacles (Cirripedia, Thoracica) of the Reykjanes Ridge // Biogeography of the North Atlantic seamounts Moscow, pp. 152–61.

[inz212389-bib-0056] Poltarukha OP , Zevina GB (2006b). Barnacles (Cirripedia, Thoracica) of the north‐eastern Atlantic // Biogeography of the North Atlantic seamounts Moscow, pp. 162–76.

[inz212389-bib-0057] Rakociński M (2011). Sclerobionts on Upper Famennian cephalopods from the Holy cross mountains, Poland. Palaeobiodiversity and Palaeoenvironments 91, 63–73.

[inz212389-bib-0058] Rakús M , Žítt J (1993). Crinoid encrusters of ammonite shells (Carixian, Tunisia). Geobios 15, 317–29

[inz212389-bib-0059] Rasmussen KA , Brett CE (1985). Taphonomy of Holocene cryptic biotas from St. Croix, Virgin Islands: Information loss and preservational biases. Geology 13, 551–3.

[inz212389-bib-0060] Ritterbush KA , Hoffmann R , Lukeneder A , De Baets K (2014) Pelagic palaeoecology: the importance of recent constraints on ammonoid palaeobiology and life history. Journal of Zoology 292, 229–41.

[inz212389-bib-0061] Roux M (1991). La Nouvelle‐Caledonie et ses alentours. Cadre géologique et océanographique du programme ENVIMARGES et de la campagne CALSUB. Documents et Travaux, Institut Géologique Albert‐de-Lapparent 15, 22–36.

[inz212389-bib-0062] Santos A , Mayoral EE , Muñiz F (2005). Bioerosion scars of acorn barnacles from the southwestern Iberian Peninsula, upper Neogene. Rivista Italianadi Paleontologia e Stratigrafia 11, 181–89 2 pls.

[inz212389-bib-0063] Saunders WB, Landman NH, eds (1987). Nautilus: The Biology and Paleobiology of a Living Fossil. Plenum Press, New York and London, 632 pp.

[inz212389-bib-0064] Saunders WB , Ward PD (2010). Ecology, distribution, and population characteristics of *Nautilus* In: Saunders WB, Landman NH, eds. Nautilus: The Biology and Paleobiology of a Living Fossil. Topics in Geobiology 6, 137–62.

[inz212389-bib-0065] Schmid–Röhl A , Röhl HJ (2003). Overgrowth on ammonite conchs: environmental implications for the Lower Toarcian Posidonia Shale. Palaeontology 46, 339–52.

[inz212389-bib-0066] Seilacher A (1960). Epizoans as a key to ammonoid ecology. Journal of Paleontology 34, 189–93.

[inz212389-bib-0067] Seilacher A (1968). Swimming habits of belemnites–Recorded by boring barnacles. Palaeogeography, Palaeoclimatology, Palaeoecology 4, 279–85.

[inz212389-bib-0068] Seilacher A (1982) Ammonite shells as habitats in the Posidonia Shale‐Floats or benthic islands?Neues Jahrbuch für Geologie und Paläontologie, Monatshefte, pp. 98–114.

[inz212389-bib-0069] Seuss B , Hembree D , Wisshak M *et al* (2015). Taphonomy of deep marine versus backshore collected *Nautilus macromphalus* conchs (New Caledonia). Palaios 30, 503–13.

[inz212389-bib-0070] Seuss B , Wisshak M , Mapes RH *et al* (2015). Syn-vivo bioerosion of *Nautilus* by endo‐and epilithic foraminiferans (New Caledonia and Vanuatu). PLoS ONE 10, e0125558. 25894584 10.1371/journal.pone.0125558 PMC4404336

[inz212389-bib-0071] Sowerby GB (1849). Monograph of the genus Nautilus. Thesaurus Conchyliorum 2, 463–5.

[inz212389-bib-0072] Suzuki H , Hayasaka S (1988). Notes on the epifauna the shells of living *Nautilus* from Fiji. Kagoshima University Research Center for the South Pacific, Occasional Papers 15, 56–9.

[inz212389-bib-0073] Tomašových A , Schlögl J , Kaufman DS *et al* (2016). Temporal and bathymetric resolution of nautiloid death assemblages in stratigraphically condensed oozes (New Caledonia). Terra Nova 28, 271–8.

[inz212389-bib-0074] Tomašových A. Schlögl J , Biroň A *et al* (2017). Taphonomic clock and bathymetric dependence of cephalopod preservation in bathyal, sediment‐starved environments. Palaios 32, 135–52.

[inz212389-bib-0075] Utinomi H (1968). A revision of the deep‐sea barnacles *Pachylasma* and *Hexelasma* from Japan, with a proposal of new classification of the Chthamalidae (Cirripedia, Thoracica). Publications of the Seto Marine Biological Laboratory 16, 21–39.

[inz212389-bib-0076] Ward PD (1987). The Natural History of Nautilus. Allen & Unwin, Boston, 267 p.

[inz212389-bib-0077] Ward PD , Martin AW (1980). Depth distribution of *N. pompilius* in Fiji and *N. macromphalus* in New Caledonia. The Veliger 22, 259–64.

[inz212389-bib-0078] Wyse Jackson PNW , Key MMK Jr (2014). Epizoic bryozoans on cephalopods through the Phanerozoic: A review. Studi Trentini Di Scienze Naturali 94, 283–91.

[inz212389-bib-0079] Yacobucci MM (2018). Postmortem transport in fossil and modern shelled cephalopods. PeerJ 6, e5909. 30515355 10.7717/peerj.5909 PMC6266924

[inz212389-bib-0080] Young PS (2002). The Verrucidae (Crustacea, Cirripedia) from the Western Coast of North America, With a Revision on the Genus Altiverruca. Arquivos do Museu Nacional (Rio de Janeiro) 60, 5–40.

[inz212389-bib-0081] Zell P , Beckmann S , Stinnesbeck W (2014). *Liostrea roemeri* (Ostreida, Bivalvia) attached to Upper Jurassic ammonites of northeastern Mexico. Palaeobiodiversity and Palaeoenvironments 94, 439–51.

